# Physiotherapy in upper abdominal surgery – what is current practice in Australia?

**DOI:** 10.1186/s40945-017-0039-3

**Published:** 2017-08-15

**Authors:** Shane Patman, Alice Bartley, Allex Ferraz, Cath Bunting

**Affiliations:** School of Physiotherapy, The University of Notre Dame Australia, Fremantle, Australia

**Keywords:** Upper abdominal surgery, Physiotherapy, Mobilisation, Ambulation, Post-operative pulmonary complications

## Abstract

**Background:**

Upper abdominal surgery (UAS) has the potential to cause post-operative pulmonary complications (PPCs). In the absence of high-quality research regarding post-operative physiotherapy management, consensus-based best practice guidelines formulated by Hanekom et al. (2012) are available to clinicians providing recommendations for post-UAS treatment. Such best practice guidelines have recommended that physiotherapists should be using early mobilisation and respiratory intervention to minimise risk of PPCs. However, recent evidence supports the implementation of mobilisation as a standalone treatment in PPC prevention, though the diversity in literature poses questions regarding ideal current practice. This project aimed to document and report the assessment measures and interventions physiotherapists are utilising following UAS, establishing whether current management is reflective of best practice guidelines and recent evidence.

**Results:**

An online survey was completed by 57 experienced Australian physiotherapists working with patients following UAS (35% survey response rate, 63% completion rate). On day one following UAS, when a patient’s condition is not medically limited, most physiotherapists routinely mobilise. Additionally, routine chest treatment continues to be implemented, with only 23% (*n* = 11/47) of physiotherapists mobilising patients without accompanying specific respiratory intervention. Variability of screening tools used to identify post-operative patients at high risk of PPC development was evident. Patient-dependent factors such as ‘fatigue’ and ‘non-compliance’ were among those identified as barriers to treatment, all influencing the commencement of treatment.

**Conclusions:**

Physiotherapists indicated that early mobilisation away from the bedside was the preferred post-operative treatment within the UAS patient population. Many continue to perform routine respiratory interventions despite recent literature suggesting it may provide no additional benefit to preventing PPCs. Current intervention choice is reflective of guidelines [1], however, recent literature has called this into question and more research needs to be done to establish if these recommendations are the most effective at reducing PPCs. Continued research is necessary to promote translation of knowledge to ensure physiotherapists are mobilising patients day one post-UAS. Likewise, future work should focus on identification of barriers, the strategies used to overcome limitations and the creation of a reliable and validated screening tool to ensure appropriate prioritisation and allocation of physiotherapy resources within the UAS patient population.

**Electronic supplementary material:**

The online version of this article (doi:10.1186/s40945-017-0039-3) contains supplementary material, which is available to authorized users.

## Background

Upper abdominal surgery (UAS) initiates a cascade of pathophysiological responses, potentially causing post-operative pulmonary complications (PPCs). There is no one definition of PPCs universally accepted in the UAS population. Surgical duration, anaesthesia and nociception impair respiratory function, exacerbate mucociliary clearance depression and suppress the cough reflex leading to secretion retention and reduced lung volumes, thereby contributing to atelectasis and the development of infection [2, 3]. Furthermore, patient dependent factors such as readiness to participate and anxiety levels, along with post-operative influences including pain, create significant barriers to treatment and promote PPC development [4, 5].

There is no published consensus around the optimal assessment tool(s) to screen patients for risk of PPC development and/or to evaluate the effectiveness of physiotherapeutic treatment post-UAS. Similarly, consensus on intervention effectiveness is currently absent, with recent research unable to demonstrate any physiotherapy technique to be superior than another at preventing PPCs [6]. Physiological outcomes of deep breathing exercises (DBEx) are variable, with many concluding that a reduction in respiratory capacity occurs regardless of prophylactic DBEx treatment [2]. There is insufficient evidence to suggest any significant clinical effectiveness of respiratory interventions such as incentive spirometry and continuous positive airway pressure post-operatively for PPC prevention [7, 8]. In comparison, recent evidence suggests that early post-operative mobilisation is a sufficient standalone treatment for patients following UAS and does not require respiratory interventions to further reduce PPCs [1, 9]. Variations in evidence and patient presentation can lead clinicians to attend to patients post-operatively based on clinical experience and observation, making it increasingly difficult to recognise what current standard physiotherapy practice is within this post-UAS population.

In the absence of high quality research and ongoing uncertainties surrounding the role and effectiveness of specific post-operative physiotherapy interventions, an international panel of experts formulated best practice recommendations for physiotherapy management within the UAS cohort [1]. Although being a lower form of evidence, the consensus recommendations suggest that the treatment parameters endorsed have reasonable generalisability across UAS patient populations; however, this is only valid if the guidelines are implemented into clinical practice.

Current post-operative UAS physiotherapy management within Australia has not been clearly documented. Therefore, this project aimed to document and report the assessment measures and interventions physiotherapists in Australia are utilising following UAS. Further, it aimed to establish whether current management was reflective of best practice recommendations as documented by Hanekom et al. [1], and demonstrate if research is being transferred and implemented into clinical practice.

## Methods

### Design

A novel, anonymous, online survey was designed to explore current practice amongst physiotherapists treating patients following UAS in general surgical wards in Australian hospitals. Closed questions were predominately used incorporating a ranking system and matrix scale; open questions and free text boxes were also included. The survey was comprised of seven (7) sections, with a total of fifty-five (55) questions aiming to investigate the assessment tools and interventions commonly used by physiotherapists in the UAS cohort.

For the purpose of this study PPC was defined as “an identifiable disease or dysfunction that is clinically relevant and adversely affects the clinical course” [1]. Likewise, risk factors that prompt PPCs in patients post-UAS were classified as per those of Souza Possa et al. [4] including advanced age, smoking history and impacts of surgery. Early mobilisation tasks were examined, which included walking away from the bedside (greater than five metres) in conjunction with upright positions, sitting out of bed and stairs [1, 10]. Definitions of frequently used terms included in the matrix styled questions were provided and designed to guide participants to answer accurately (see Additional file 1). Other definitions of commonly used terms were also supplied to participants.

### Ethics approval

The study was approved by the Human Research Ethics Committee (HREC) at The University of Notre Dame Australia (015151F) and by relevant local institutional HRECs.

### Piloting

Prior to use, four independent individuals piloted the survey for readability and face validity; three were experienced cardiorespiratory physiotherapists with a particular interest and knowledge regarding UAS, the fourth was a health professional in a different field of work. Piloting identified any unanticipated problems and ambiguity within the instructions and questions and recognised time commitments required to complete the survey, allowing modification prior to dissemination. Minor amendments only were identified requiring adjustment for enhancing clarity on a couple of questions, plus some formatting adjustments of the online tool to enhance presentation / readability, and the online survey was finalised for distribution in February 2016.

### Participants

Participants were included if they were qualified physiotherapists treating patients following UAS in an Australian hospital.

### Recruitment

All Australian hospitals that conducted general surgery were identified via publicly accessible websites. Hospitals were contacted to establish if UAS was performed and whether the facility provided a physiotherapy service to patients undergoing UAS.

Hospital contact details were distributed between researchers by a random sequence generator in order to avoid bias. Phone calls were directed to the Physiotherapy Head of Department who provided further contact details and/or email addresses of physiotherapists. An outline of the study’s objectives was discussed during the phone calls, emphasising the necessity for physiotherapists treating patients undergoing UAS to be involved. Participants were encouraged to forward on the email to other relevant clinicians, increasing response rate via the snowball effect.

### Sample size

As this study was descriptive and did not test any hypotheses, no sample size calculations were undertaken. A purposeful sample of convenience was used. All hospitals performing UAS were targeted resulting in a total population of 189 Australian hospitals being contacted. Not all facilities performed UAS or had physiotherapists treating these patients, whilst others did not want to provide contact details due to security and/or confidentiality reasons. As a result, contact details of 178 physiotherapists were retrieved.

### Distribution

An invitation to participate with the anonymous survey was distributed by email in February 2016 using the online survey tool Qualtrics (http://www.qualtrics.com). Voluntarily opening and completing the survey implied consent.

Participants were given up to 3 months to complete the survey; follow up emails were sent to participants prompting completion of the survey at four, 6 and 8 weeks after the initial email was sent, aiming to optimise response rate.

### Data analysis

Data was anonymously collected via the Qualtrics Q-Lite Package then exported into Microsoft Excel (Version 1.23.1 for Mac) for analysis. Categorical data were expressed in terms of count, frequency and proportions, primarily reporting percentages and means, specifically clarifying the total responses (n). Means were used to decipher the matrix styled questions. A 5-point Likert scale was used, aligning with “never” to “always”. For ranking styled questions, respondents were limited to three responses, allowing greater control of bias. Quantitative content analysis was used to determine patterns of participant responses to open ended questions, these were organised using a category matrix describing post-operative treatment goals [11]. Frequency counts were undertaken and expressed as numbers and percentages.

## Results

**Fig. 1:**
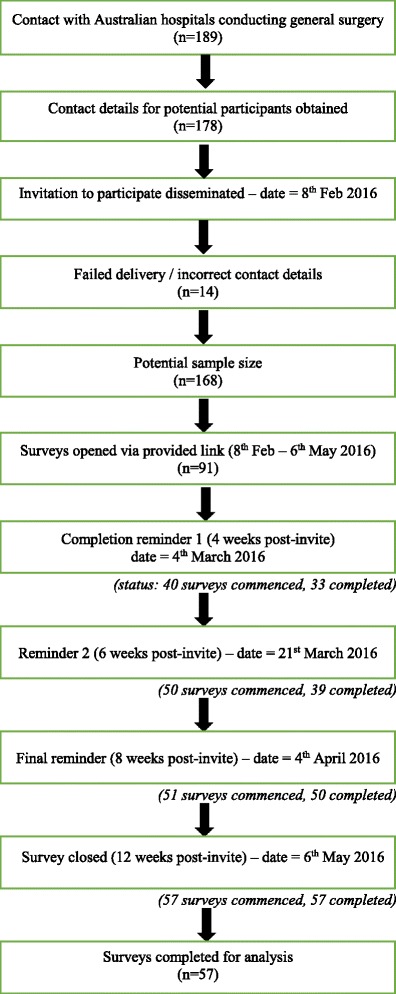
Participant flowchart

A total of 178 email invites were sent, of which 14 failed delivery, and 91 survey links were opened, providing a consenting rate of 56%. Of these 91 surveys successfully distributed and opened, 57 were completed (35% survey response rate; 63% completion rate), with the remaining 34 potential participants opening and viewing the survey but not progressing to engage to leave responses. Fig. 1 provides a flowchart of participant recruitment. Question responses were not forced in order to proceed through the online survey; consequently not all 57 respondents completed every question, necessitating reporting “n” per question. Due to low variance across the Likert scale, to facilitate interpretation of data obtained responses of “never” and “rarely” were collapsed and reported together, as were “always” and “often”, whilst “sometimes” remained the same, thereby resulting in three groupings for reporting. Tables 3 -6 provide the original 5-point scale data.

### Demographics

Demographics of participants and hospitals shown in Table 1.

**Table 1:** Participant and hospital demographics (*n* = 57). Data are expressed as number (%) unless otherwise specified

Demographics		
Age - years [mean (range)]		35 (21–63)
Gender	Female	45 (79)
State	VIC	19 (33)
	NSW	11 (19)
	WA	11 (19)
	ACT/NT/TAS	6 (11)
	QLD	5 (9)
	SA	5 (9)
Years of experience as a physiotherapist	1–5 years	15 (26)
	6–10 years	16 (28)
	>10 years	26 (46)
Years of experience in General Surgical Ward	<1 year	7 (12)
	1–2 years	9 (16)
	3–5 years	6 (11)
	>5 years	35 (61)
Hospital^a^	Public	39 (68)
	Private	16 (28)
	Mixed	2 (4)
Type^a^	Tertiary	9 (16)
	Secondary	2 (4)
Setting^a^	Metropolitan	16 (28)
	Regional	6 (11)
Number of beds in General Surgical Wards	0–10	4 (7)
	10–20	3 (5)
	> 20	50 (88)

^a^question = “What type of hospital do you currently work at? Please select all that apply.” multiple incomplete responses per option, therefore % not equal to 100

*ACT* Australian Capital Territory, *NSW* New South Wales, *NT* Northern Territory, *QLD* Queensland, *Tas* Tasmania, *WA* Western Australia, *VIC* Victoria, *SA* South Australia

Hospital: refer to - http://www.aihw.gov.au/haag12-13/public-and-private-hospitals/

Type: refer to - http://healthissuescentre.org.au/consumers/health-care-in-australia/understanding-our-health-care-system

### Screening patients for pre-existing and post-operative risk factors prior to commencing treatment

Screening of patients prior to UAS by physiotherapists was not performed routinely; 51% respondents (*n* = 29/57) reporting this was “never” part of their practice, with a further 37% (*n* = 21/57) responding that they “rarely” screened / assessed patients prior to their UAS.

From a list provided to them, physiotherapists were asked to specify the frequently used parameters they utilise to assist them in identifying pre-existing risk factors that put patients at greater risk of PPC development. ‘Advanced age’ and ‘smoking history’ were both recognised by 98% (*n* = 54/55) of respondents as pre-existing risk factors; 94% (*n* = 52/55) of respondents noted that ‘pre-morbid respiratory conditions’ are “always” a risk factor, with 91% (*n* = 50/55) agreeing that ‘pre-existing heart conditions’ were also a factor to acknowledge. Screening tools “never” used include ‘neurological conditions’ (53%, *n* = 29/55) and an ‘American Society of Anesthesiology (ASA) score greater than 2’ (42%, *n* = 23/55).

Post-operatively, physiotherapists commonly use ‘chest x-ray’ (81%, *n* = 21/26), ‘auscultation’ (77%, *n* = 20/26) and ‘decreased saturation of oxygen (SpO_2_)’ (77%, *n* = 20/26) as parameters to screen for high priority patients. ‘Surgical duration’ was rated by 58% (*n* = 32/55) as “sometimes” and “often” by 12% (*n* = 12/55) as being used to identify a patient at risk of PPCs. However, respondents did not rate ‘sputum classification’ (46%, *n* = 12/26) or ‘high temperature’ (50%, *n* = 13/26) as commonly used post-operative screening tools.

### Treatment

Physiotherapists were asked to comment on their primary goals and/or foci for their management of patients over consecutive days post-UAS. Recurring statements indicated the majority of patients were not seen on day zero (day of surgery). On average, 94% (*n* = 51/54) of physiotherapists in general surgical wards treated their UAS patients once daily, with 93% (*n* = 51/55) initiating intervention on day one post-operatively.

On day one post-operatively, 85% (*n* = 40/47) indicated their goal was to mobilise their patients away from the bedside, with all physiotherapists expecting patients to achieve transferring bed to chair to sitting out of bed (SOOB) and only 11% (*n* = 5/47) suggesting their focus was for patients to be SOOB without further mobilising. Physiotherapists’ expectations of patient milestones achieved each consecutive day post-UAS are given in Table 2.

**Table 2:** Milestones expected to be achieved following UAS

*(n = 45)*	Day 1	Day 2	Day 3–5
Bed exercises	78	20	13
Sitting over edge of bed	**98**	18	18
Transferring bed to chair	**100**	24	22
Marching on spot (at bedside)	**96**	29	18
Walking away from bed (5+ m) with assistance	**80**	47	22
Walking independently with gait aid away from bed	20	67	58
Walking independently without gait aid away from bed	9	31	96
Stairs	2	18	96
Other(s) (please specify)	4	7	13

Values expressed as % where participants answered “always”. Values in bold indicate the top 4 expected milestones for day 1

The following comment reflects the diversity of a potential physiotherapy treatment day one:

*“Initial assessment and identifying main issues. Education +++, strategies to reduce pain during transfer to sit on edge of bed, aim to sit out of bed, formal prescription/completion of deep breathing/bubble PEP/incentive spirometry to increase ventilation. Sputum clearance as required with supported huff/cough. Wean oxygen as able. If physiologically responding well to all of the above, trial short walk with aid.”*

Consistent with their stated goals, physiotherapists are almost universally prescribing mobilisation away from the bedside as their primary treatment day one, as evident by 87% (*n* = 40/47) either “often” or “always” selecting ‘ambulation’ (Table 3). When asked what they typically included in a physiotherapy prescribed mobility program, the 35 free text responses can be globally summarised as incorporating graded/progressive walking and functional activities, with consideration of frequency, intensity and duration, with an education component. Respiratory interventions (such as deep breathing exercises and/or supported cough were consistently prescribed within the first 3 days post-operatively. Physiotherapists were asked if they prescribed respiratory interventions to patients post-operatively, of which 93% (*n* = 43/46) of physiotherapists said “always”. Seventy-seven per cent (*n* = 36/47) reported that they had aimed to incorporate chest treatment as standard practice, and 23% (*n* = 11/47) commented that mobilisation without specific respiratory intervention was their primary aim of treatment over the consecutive days.

**Table 3:** Frequently prescribed and used interventions over consecutive days post-UAS

	DAY 1					DAY 2					DAY 3				
***(n = 46)***	Never	Rarely	Sometimes	Often	Always	Never	Rarely	Sometimes	Often	Always	Never	Rarely	Sometimes	Often	Always
DBEx	0	4	9	**48**	**39**	0	7	22	39	33	4	22	30	28	15
ACBT	4	22	30	30	13	4	26	33	28	9	11	24	43	17	4
FET	2	15	35	22	26	2	20	37	24	17	7	28	37	22	7
Cough	43	41	11	4	0	41	41	13	4	0	35	39	20	7	0
Supported cough	0	0	7	**33**	**61**	0	0	9	**35**	**57**	0	2	30	**28**	**39**
Cough assist	67	17	13	0	2	65	20	13	0	2	72	20	7	0	2
Suction	17	57	24	2	0	20	67	13	0	0	24	67	9	0	0
PEP	13	37	41	9	0	15	37	43	4	0	22	41	33	4	0
CPAP	48	43	9	0	0	50	46	4	0	0	57	43	0	0	0
IS	41	37	15	4	2	43	35	15	4	2	54	30	11	2	2
Aerosol therapy	11	33	50	7	0	11	30	54	4	0	15	41	41	2	0
Bed mobility	0	17	15	26	41	2	24	11	30	33	7	26	20	17	30
Upright/SOOB	0	4	9	30	57	0	4	9	**26**	**61**	2	13	9	**22**	**54**
Sit to stand	0	0	4	**35**	**61**	0	2	2	**28**	**67**	0	7	7	**17**	**70**
Ambulation	0	0	13	**28**	**59**	0	0	0	**24**	**76**	0	0	2	**20**	**78**
Stairs/steps	43	33	17	4	2	11	30	39	15	4	0	9	35	39	17
Cycle pedals	65	26	9	0	0	57	33	11	0	0	52	35	11	2	0
UL exercises	13	28	39	13	7	11	28	37	17	7	13	24	39	17	7
Education	0	0	7	**17**	**76**	0	2	7	**13**	**78**	0	4	4	15	76

Values expressed as %. Values in bold represent frequently prescribed and used interventions for particular days

*DBEx* deep breathing exercises, *ACBT* active cycle of breathing, *FET* forced expiratory techniques, *PEP* positive expiratory pressure, *IS* incentive spirometry, *CPAP* continuous positive airway pressure, *SOOB* sitting out of bed, *UL* upper limb

The choice of respiratory components incorporated into post-operative treatment was variable amongst physiotherapists (Table 4). The most commonly prescribed component was a huff +/− cough, followed by positioning, thoracic expansion exercises and sustained maximal inspirations; however, no component was prescribed significantly more than another.

**Table 4:** Components of breathing exercises

*(n = 46)*	Never	Rarely	Sometimes	Often	Always
Inspiratory hold	4	4	41	41	9
SMI	9	22	24	37	9
TEE	2	13	26	**48**	**11**
Breathing control	2	20	48	26	4
Huff +/− cough	0	0	20	**39**	**41**
Proprioceptive facilitation	7	15	46	30	2
Positioning	0	9	13	**39**	**39**
Rib springing concept	46	37	15	2	0
PLB	17	35	39	9	0
PEP	7	30	46	17	0
CPAP	37	41	22	0	0
IS	41	35	15	7	2
IPPB	70	24	7	0	0
Other(s) (please specify)	83	9	7	0	2

Values expressed as %. Values in bold represent the most frequently used components of breathing exercises

*SMI* sustained maximal inspiration, *PEP* positive expiratory pressure, *IS* incentive spirometry, *TEE* thoracic expansion exercises, *CPAP* continuous positive airway pressure, *IPPB* intermittent positive pressure breathing, *PLB* pursed lip breathing

### Outcome measures

Physiotherapists identified ‘distance’, ‘progression of assistance required’, ‘readiness for discharge’ and ‘SpO_2_’ as the key outcome measures used to evaluate effectiveness of their intervention, with the ‘BORG score’ [12] and ‘Clinical Pulmonary Infection Score (CPIS)’ [13] being used infrequently (Table 5).

**Table 5:** Outcome measures used to monitor effectiveness of interventions

*(n = 46)*	Never	Rarely	Sometimes	Often	Always
Respiratory rate	2	13	35	39	11
FiO_2_/O_2_ requirements	4	7	9	59	22
SpO_2_	0	2	11	50	**37**
Chest x-ray	2	7	52	37	2
Auscultation	0	2	15	50	**33**
Clinical Pulmonary Infection Score (CPIS)	**72**	22	2	0	4
Sputum clearance	0	4	24	48	24
ABGs	**13**	17	46	20	4
Blood pressure	4	50	20	24	2
Heart rate	7	37	26	26	4
Pain (VAS)	2	20	37	26	15
Rate of Perceived Exertion (RPE)	**13**	30	33	22	2
BORG score	**20**	35	28	15	2
Progression of assistance required	0	4	4	57	**35**
Distance mobilised	0	0	7	48	**46**
Readiness for discharge	0	0	13	57	30
Anxiety level	2	30	39	26	2
Patient appearance	2	17	33	30	17
Other(s) (please specify)	85	2	9	2	2

Values expressed as %. Values in bold represent the top 4 “always” and “never” used outcome measures

*FiO*_*2*_ fraction of inspired oxygen, *SpO*_*2*_ oxygen saturation, *ABGs* arterial blood gases, *BORG* Borg Scale, *VAS* visual analogue scale

### Barriers to treatment

When physiotherapists provided their opinion regarding barriers to commencing intervention post-operatively, no option scored highly in the “always” category. ‘Pain’ was the most common patient-dependent barrier, followed by ‘blood pressure’, ‘patient readiness’ and ‘fatigue’. In comparison, physiotherapists agreed that general care factors including ‘physician instruction’ and ‘availability of staff and equipment’ only occasionally influenced commencement of their treatment (Table 6). Factors most likely to interfere with the frequency of structured mobility on each consecutive day were ‘patient condition’ (88%, *n* = 37/42), ‘staff availability’ (50%, *n* = 21/42) and ‘patient compliance’ with physiotherapy (48%, *n* = 20/42).

**Table 6:** Factors limiting commencement of physiotherapy intervention

*(n = 54)*	Never	Rarely	Sometimes	Often	Always
Patient limiting factors					
Pain (high VAS)	2	13	46	39	0
Level of ventilation/O_2_ requirements	11	46	35	7	0
Decreased SpO_2_	19	46	31	4	0
VO_2_ max	54	39	7	0	0
FiO_2_	20	54	22	4	0
Presence of spontaneous breathing	30	48	19	4	0
Abnormal respiratory rate	13	56	31	0	0
Reduced exercise tolerance/fitness	19	41	33	7	0
BORG	26	54	19	2	0
Blood pressure	4	13	59	24	0
Abnormal heart rate	7	39	43	9	2
ABGs	24	56	15	6	0
Number of attachments (catheter, IV drip, O_2_ therapy)	41	48	7	4	0
BMI	24	56	17	4	0
Patient readiness	7	28	43	19	4
Anxiety level of patient	6	33	43	17	2
Physio judgement of medical stability	11	20	48	17	4
Fatigue	11	26	54	7	2
Other(s) please specify	78	7	13	2	0
General limiting factors					
Physician instructions	7	35	46	9	2
Assistance required (mobility)	24	35	30	11	0
Availability of equipment	24	41	20	15	0
Availability of staff	15	31	37	15	2
Pressure to discharge from ward	20	41	31	6	2
Conflicts with MDT appointments	19	54	26	2	0
Other(s) (please specify)	87	6	7	0	0

Values expressed as %

*VAS* visual analogue scale, *O*_*2*_ oxygen, *BMI* body mass index, *IV* intravenous, *BORG* Borg Scale, *SpO*_*2*_ oxygen saturation, *ABGs* arterial blood gases, *VO*_*2*_ *max* maximal oxygen uptake, *FiO*_*2*_ fraction of inspired oxygen, *MDT* multi disciplinary team

## Discussion

This study documents current post-operative physiotherapy management of patients following UAS.

Despite not seeing patients pre-operatively, physiotherapists currently undertake post-operative screening utilising a variety of assessment tools, and treat patients with a combination of early mobilisation and respiratory interventions post-operatively. The mean age of participants was 35 years, with the majority of respondents having practiced physiotherapy for greater than 10 years, with more than 5 years’ experience in general surgical wards. This provides confidence that the received responses are from highly experienced physiotherapists with considerable knowledge and experience within UAS practice.

### Screening patients for pre-existing and post-operative risk factors prior to commencing treatment

#### Pre-existing risk factor screening

This study identified that the majority of physiotherapists surveyed do not currently perform routine pre-operative screening or interventions on patients prior to their UAS. Early reports from the LIPSMAck POP trial [14] suggest that pre-operative interventions have the potential to positively influence patient outcomes post-operatively. This indicates that a significant change in practice will need to be undertaken across Australia in order to ensure the key research findings are translated into practice. Improved access to patients pre-operatively will also provide opportunities for pre-operative screening. This encourages the identification of high-risk patients, allowing them to be prioritised post-operatively, ensuring the best allocation of physiotherapy resources and a potential to further reduce PPC rates.

Despite not pre-operatively screening patients, Australian physiotherapists are assessing patients early post-operatively for pre-existing risk factors for PPC development. Physiotherapists used advanced age, respiratory and cardiac co-morbidities, and smoking history as primary pre-existing parameters to screen whether their patient was at high risk of a PPC, with the majority of respondents suggesting that past respiratory history was a factor related to PPCs. These factors are reflective of those described by Haines et al. [10] and Scholes et al. [15], and assist clinicians’ ability to screen for high-priority patients that are at greater risk of PPC development in the post-operative period. This is important to ensure physiotherapy interventions are allocated and targeted to those who are most likely to benefit [15].

Coincidently, the risk factors commonly identified by clinicians equate to an ASA score greater than two [15]. Despite this, use of the ASA scoring system as a screening tool was not common amongst respondents. This could indicate a lack of awareness of the ASA scoring system as a well-documented, validated assessment and predictive tool for PPCs, or that physiotherapists do not regard it as relevant to their practice. Additionally, clinicians failed to comment on other factors used to screen patients, such as pre-operative exercise capacity and pre-existing neurological conditions. This is despite them being identified as having an impact on respiratory function and patient outcomes post-operatively [15].

#### Post-operative risk factor screening

The development of diagnostic criteria specific to UAS (such as those of Scholes et al. [15]) assists physiotherapists in the identification of variables that place patients at higher risk of PPC development post-operatively. Clinicians did not identify ‘high temperature’ or ‘sputum classification’ as frequently used screening tools, despite being recommended measures to identify infection post-operatively [15]. Despite its potential to reduce mobility, ‘non-compliance’ did not prove to be an indicator used by physiotherapists to recognise someone at high-risk of complications. Likewise, clinicians did not recognise ‘duration of surgery and anaesthesia’ as influences to PPC development. Nevertheless, physiotherapists identified ‘chest x-ray’, ‘auscultation’ and ‘decreased SpO_2_’ as common screening tools which reflect those mentioned by Scholes et al. [15].

Various diverse screening tools are being used throughout clinical practice to identify a patient at risk of developing a PPC. This calls for additional work to form an agreed consensus on the key assessment tools available to clinicians within the UAS patient population. This is likely to improve physiotherapists’ efficiency at screening and prioritising treatment to high-risk patients, reducing the severity and impact of PPCs, and allowing for the appropriate allocation of resources [15].

### Respiratory intervention

Results from this survey suggest that physiotherapists are currently implementing respiratory interventions into their practice; more than half combining chest treatments and mobilisation as their standard practice. This is despite recent evidence supporting the use of mobilisation as a standalone treatment, concluding that the addition of DBEx and coughing provides no additional benefit [9].

The majority of respondents specified an aim to perform routine chest treatment on day one, with results indicating that physiotherapists universally prescribed DBEx and supported coughs. It is evident that positioning and TEEs are also favourable interventions, with over half of clinicians consistently implementing them. These results are reflective of Hanekom et al. [1] who recommended that respiratory interventions are warranted for patients post-UAS. These recommendations are purely based on clinical experience, as the current literature remains somewhat inconclusive. A further study found adherence to mobilisation and chest therapy was effective at reducing the incidence of atelectasis to 0% [4], but continued research is necessary to validate this claim.

Despite conflicting evidence, clinical experience may be the primary driver behind why physiotherapists continue to use chest treatment as standard practice and not as per required. Although clinical experience is not necessarily unreliable, it needs to be acknowledged as a potential factor in resistance to change and should be integrated with evidence from high quality studies to promote best practice for patients undergoing UAS.

Further research to clarify the role of standard respiratory interventions and translation of evidence-based practice within UAS has the potential to encourage physiotherapists to agree and consistently implement interventions that are validated and most beneficial to this patient population, whilst best utilising valuable physiotherapy resources. That being said, there was no indication throughout this study that physiotherapists were using respiratory techniques as a standalone treatment, as mobilisation was universally accepted as the optimal choice of treatment in this patient population.

### Mobilisation

Hanekom et al. [1] reported early mobilisation to be a beneficial intervention for patients following UAS. This is further validated by Silva and colleagues [9] emphasising the benefits of early mobilisation away from the bedside when performed at sufficient intensities, whilst Haines et al. [10] established that delaying early mobilisation caused an increase in PPCs.

This study demonstrated a positive link between a majority of milestones clinicians expected patients to achieve post-operatively and the physiotherapy treatment actually delivered over consecutive days. Following day one, physiotherapists indicated that when their patients were medically stable, providing no limitations to physiotherapy management, they mobilised their patients away from the bedside. This is in line with physiotherapists’ expectations and primary focus that from day one onwards, all patients should be mobilising away from the bedside. These findings are similar to the pre-existing literature of Silva et al. [9] and Haines et al. [10] concluding that the implementation of mobilisation alone provided an adequate reduction in PPC rates. This suggests recent literature is being translated into current practice as Australian physiotherapists demonstrated an awareness of mobilising away from the bedside as an effective treatment post-operatively.

Stair climbing was not necessarily being prescribed as an intervention despite physiotherapists expecting patients to achieve it as a milestone. Respondents also indicated that cycle pedals are an uncommon intervention post-operatively despite Bhatt and colleagues [6] determining that early aerobic exercise through the use of cycle pedals halved the rate of respiratory infection and length of stay. This was, however, a small single-centre study that needs to be validated prior to translation into standard physiotherapy practice in upper abdominal surgery patients.

Overall, this study’s results suggest that physiotherapists are implementing early mobilisation and that it reflects recent literature within this patient population. Despite all physiotherapists identifying early mobilisation as the primary focus of treatment, a small percentage of physiotherapists acknowledged that it was not implemented on every occasion, suggesting that barriers to ideal treatment exist.

### Barriers to treatment

Findings of this survey indicate that a variety of patient-dependent factors limit the commencement of physiotherapy treatment post-operatively. ‘Pain’ was the most prominent barrier reported, followed by ‘fatigue’ and ‘patient readiness’, all having the capacity to reduce mobility and hence increasing the risk of a PPC. Similarly, ‘non-compliance’ was an evident barrier to treatment despite not previously being recognised as a post-operative risk factor for PPC development and delayed mobility.

The barriers identified by physiotherapists in this study are reflective of those previously reported by Browning et al. [16], in particular ‘availability of staff’ and ‘assistance to mobilise’ were both found to affect the amount of ‘uptime’ patients receive post-operatively following UAS. ‘Patient condition’ and ‘patient compliance’ were also reported as factors impacting the commencement and frequency of treatment, especially mobility, in this study.

Strong, validated evidence could give physiotherapists the opportunity to become more autonomous in the prescription of interventions post-operatively, assisting them to overcome external barriers such as ‘physician instruction’. There could be additional value in finding ways to support knowledge translation beyond physiotherapy cohorts and outwards to the wider field of the multi-disciplinary team to enhance physiotherapy management of patients post-UAS.

Barriers to treatment was not a focus of this study, therefore as a consequence of these incidental findings, it is unclear as to whether barriers such as pain and fatigue limit the efficiency and desired outcome of physiotherapy interventions or if it prevents the commencement of treatment completely. These findings provide avenue for further investigation into the impact of these barriers on commencing treatment and the strategies physiotherapists use to overcome them, creating the foundation of future studies to discover ways to facilitate treatment.

### Limitations

Despite piloting, the length of the survey was the primary limitation of this study, with only two-thirds of those commencing survey completing (38/57). Not all questions were universally answered, with some respondents commenting that various questions were not applicable and/or repetitive, despite such issues not being apparent with piloting. Additionally, respondents may have perceived the questions differently to what was intended, again despite face validity being a focus of the piloting. Not all question responses were mandated in order to proceed through the survey, possibly accounting for varied response rates per question; providing opportunities for ‘not applicable’ answer options may be appropriate for future projects. The survey was anonymous potentially allowing multiple people to contribute from one facility. Likewise, the rotational nature of physiotherapy jobs may have hindered the response rate. Nevertheless, reminder emails worked to increase response rates to an adequate number with desirable representation Australia wide. Useful information was obtained throughout the survey making it a reflective summary of current practice in the UAS population.

### Recommendations for future work

Continued research is necessary to determine whether the addition of respiratory interventions to early mobilisation confers any additional benefit to mobilisation alone in preventing PPCs. Further discussion is necessary to establish whether formalising an agreed minimal dataset of core screening tools could be a potential solution for prioritising resources. Also, further investigation into the barriers to treatment need to be completed. This has the potential to reduce PPC rates, improve patient-related outcomes and encourage the appropriate use of physiotherapy resources.

## Conclusion

This study found that most Australian physiotherapists are mobilising their patients away from the bedside early in the post-operative period following UAS, with many continuing to also incorporate routine respiratory interventions. The interventions currently implemented by physiotherapists for patients post-UAS are reflective of the guidelines from Hanekom et al. [1]. However, more recent evidence emphasises the use of early mobilisation as a standalone treatment [9], which was not yet reflected in current practice. The variability of screening tools used amongst clinicians to identify high-risk patients post-operatively was reflective of the scarce amount of validated evidence available to physiotherapists. In combination with future research, an agreement amongst clinicians is required to establish a baseline collection of screening tools and interventions to assist clinicians with appropriately prioritising patients following UAS to ensure physiotherapy treatment time is allocated and utilised efficiently.

## Additional file

Additional file 1: Survey tool. (PDF 401 kb)

## Abbreviations

ABGs: Arterial blood gases
ACBT: Active cycle of breathing technique
ACT: Australian Capital Territory
ASA: American Society of Anesthesiology
BMI: Body mass index
BORG: Borg Scale
CPAP: Continuous positive airway pressure
CPIS: Clinical pulmonary infection score
DBEx: Deep breathing exercises
FET: Forced expiratory techniques
FiO_2_: Fraction of inspired oxygen
HREC: Human Research Ethics Committee
IPPB: Intermittent positive pressure breathing
IS: Incentive spirometry
IV: Intravenous
MDT: Multi-disciplinary team
NSW: New South Wales
O_2_: Oxygen
PEP: Positive expiratory pressure
PLB: Pursed lip breathing
PPCs: Post-operative pulmonary complications
QLD: Queensland
SA: South Australia
SMI: Sustained maximal inspiration
SOOB: Sitting out of bed
SpO_2_: Oxygen saturation
TEE: Thoracic expansion exercises
UAS: Upper abdominal surgery
UL: Upper limb
VAS: Visual analogue scale
VIC: Victoria
VO_2 max_: Maximal oxygen uptake
WA: Western Australia
