# Supplementary material for: Physiotherapy in upper abdominal surgery – what is current practice in Australia?
Source: Arch Physiother. 2017 Aug 15;7:11. doi: 10.1186/s40945-017-0039-3 (PMC5759921; doi:10.1186/s40945-017-0039-3)
Supplement: Additional file 1: — Survey tool. (PDF 401 kb) [file 40945_2017_39_MOESM1_ESM.pdf]

THE UNIVERSITY OF NOTRE DAME AUSTRALIA

Matrix Styled Rating: Always 100%-80% Very often 80%-60% Sometimes 60%-40% Rarely 40%-20% Never 0%-20%

Participant Information

Q1. What state do you practice in?

ACT/NT/TAS NSW QLD WA VIC SA

Q2. What is your gender?

Female Male

Q3. Please indicate your age

Q4. What year did you become a registered Physiotherapist?

Q5. How many years of experience do you have working in general surgery?

Less than 1 year 1-2 years 3-5 years More than 5 years

Q6. What type of hospital do you currently work at? Please select all that apply.

Public

Private

Mixed

Secondary

Tertiary

Metropolitan

Regional

Other (please specify)

Q7. How many beds are there in the General Surgery ward(s)?

0-10 | 10-20 | 20-30 | More than 30

Q8. Approximately, how many full time physiotherapists work in the general surgical ward(s) on a standard workday during the week?

Q9. On an average working day, approximately how many patients do you see who have undergone upper abdominal surgery (UAS)?

Q10. In a standard week, what percentage of patients do you see that have undergone the following types of surgeries?

	0	10	20	30	40	50	60	70	80	90	100
Oesophagectomy											
Open gastric surgery											

Whipple's/PPPD

Other pancreatic surgery

Hepatectomy/Lobectomy

Other liver surgery

Colorectal

Other (please specify)

Patient Demographics

Q11. Do you screen/assess your patients prior to their UAS?

Never Rarely Sometimes Often Always

Q12. If yes, what do you use to screen your patients?

	Never	Rarely	Sometimes	Often	Always
Melbourne Risk Prediction Tool	○	○	○	○	○
Physician referral	○	○	○	○	○
Smoking history	○	○	○	○	○
ASA Score (American Society of Anaesthesiology)	○	○	○	○	○
Past clinical experience	○	○	○	○	○
Mobility status	○	○	○	○	○
BMI (body mass index)	○	○	○	○	○
National Surgical Quality Improvement Project (NSQIP)	○	○	○	○	○

Fitness level/exercise tolerance	○	○	○	○	○
Other(s) (please specify) ______	○	○	○	○	○

Q13. What **outcome measures (OCM)** do you use to identify a patient at high-risk of PPC development?

	Never	Rarely	Sometimes	Often	Always
Pain (high VAS)	○	○	○	○	○
Respiratory PMHx	○	○	○	○	○
Patient appearance	○	○	○	○	○
Raised blood pressure	○	○	○	○	○
High Rate of Perceived Exertion (RPE)	○	○	○	○	○
Auscultation (abnormal findings)	○	○	○	○	○
High BORG	○	○	○	○	○
High temperature	○	○	○	○	○
Chest X-Ray (abnormal findings)	○	○	○	○	○
Raised respiratory rate	○	○	○	○	○
Amount of assistance required (mobility)	○	○	○	○	○
Sputum classification	○	○	○	○	○
Increased FiO2	○	○	○	○	○
Altered ABGs	○	○	○	○	○
Non-compliance	○	○	○	○	○
Anxiety level	○	○	○	○	○

Decreased SpO2	○	○	○	○	○
Other(s) (please specify) ______	○	○	○	○	○

Q14. After surgery, when would you first see your patient?

Day of surgery (day 0)

Day after surgery (day 1)

Two (2) days after surgery

Three to five (3-5) days after surgery

Q15. What pre-existing postoperative pulmonary complication (PPC) risk factors do your UAS patients commonly present with?

	Never	Rarely	Sometimes	Often	Always
Smoking history	○	○	○	○	○
Malnutrition	○	○	○	○	○
ASA Score of >2	○	○	○	○	○
Obesity/high BMI	○	○	○	○	○
Heart conditions	○	○	○	○	○
Respiratory conditions	○	○	○	○	○
Surgical duration (5+ hours)	○	○	○	○	○
Duration of anaesthesia (>120 mins)	○	○	○	○	○
Older age (>59 yo)	○	○	○	○	○
Neurological conditions	○	○	○	○	○
Need for mechanical ventilation +/- ICU admission	○	○	○	○	○
Diabetes	○	○	○	○	○

Compromised lung volumes	○	○	○	○	○
Muscle weakness	○	○	○	○	○
Other(s) (please specify) ______	○	○	○	○	○

Patient-Physiotherapy Factors for Commencing Treatment

Q16. How frequently, on average, would a standard post-operative patient receive physiotherapy intervention?

- Never
- Once daily
- Twice daily
- Three times a day
- Hourly
- Other (please specify) ______

Q17. In your opinion, what **patient factors** limit the commencement of your intervention?

	Never	Rarely	Sometimes	Often	Always
Pain (high VAS)	○	○	○	○	○
Reduced exercise tolerance/fitness	○	○	○	○	○
Level of ventilation/O2 requirements	○	○	○	○	○
BMI	○	○	○	○	○
Blood pressure	○	○	○	○	○
Number of attachments (catheter, IV drip, O2 therapy)	○	○	○	○	○

Abnormal heart rate					
BORG					
Abnormal respiratory rate					
Decreased SpO2					
ABGs					
Patient readiness					
Anxiety level of patient					
Presence of spontaneous breathing					
VO2 max					
FiO2					
Physio judgement of medical stability					
Fatigue					
Other(s) please specify					

Q18. In your opinion, what **general care factors** limit the commencement of your intervention?

	Never	Rarely	Sometimes	Often	Always
Physician instructions					
Assistance required (mobility)					
Availability of equipment					
Availability of staff					
Pressure to discharge from ward					
Conflicts with MDT appointments					

Other(s) (please specify) ______	○	○	○	○	○

Prescription and Dosage of Interventions

Q19. What is your **primary focus/goals for physiotherapy management** over consecutive days post UAS? What do you aim to achieve?

Day of surgery (day 0)	
Day after surgery (day 1)	
Two (2) days after surgery	
Three to five (3-5) days after surgery	
Not applicable (N/A)	

Q20. If the patients condition is not limited, what **interventions** do you frequently prescribe and use over consecutive days post UAS?

	DAY 1							
	Never	Rarely	Sometimes	Often	Always	Never	Rarely	S[illegible]
Deep breathing exercises (TEE, SMI, PLB)	○	○	○	○	○	○	○	
Active Cycle of Breathing Technique (ACBT)	○	○	○	○	○	○	○	
Forced Expiratory Techniques (FET - huff + breathing control)	○	○	○	○	○	○	○	
Positive Expiratory	○	○	○	○	○	○	○	

Pressure (PEP)							
Incentive Spirometry (IS)	○	○	○	○	○	○	○
CPAP (Continuous Positive Airway Pressure)	○	○	○	○	○	○	○
Aerosol therapy (humidification, nebulisers)	○	○	○	○	○	○	○
Bed mobility (rolling, bridge etc)	○	○	○	○	○	○	○
Upright sitting on edge of bed	○	○	○	○	○	○	○
Sit to stand	○	○	○	○	○	○	○
Ambulation/walking	○	○	○	○	○	○	○
Stairs/steps	○	○	○	○	○	○	○
Upper limb exercises	○	○	○	○	○	○	○
Cough (unsupported)	○	○	○	○	○	○	○
Supported coughing	○	○	○	○	○	○	○
Suction	○	○	○	○	○	○	○
Education	○	○	○	○	○	○	○
Cycle pedals	○	○	○	○	○	○	○
Cough assist	○	○	○	○	○	○	○

Q21. What options have we missed? Please state other interventions you use.

Q22. Of the interventions listed below, rank from 1 to 3 the **interventions** you most commonly use on a standard post-op patient.
(Rank where 1 is the most commonly used intervention)

☐ Deep breathing exercises (LET, TEE, SMI, PLB)

☐ Active Cycle of Breathing Technique (ACBT)

☐ Forced Expiratory Techniques (FET - huff + breathing control)

☐ Positive Expiratory Pressure (PEP)

☐ Incentive Spirometry (IS)

☐ CPAP (Continuous Positive Airway Pressure)

☐ Aerosol therapy (humidification, nebulisers)

☐ Bed mobility (rolling, bridge etc)

☐ Upright sitting on edge of bed

☐ Sit to stand

☐ Ambulation/walking

☐ Stairs/steps

☐ Upper limb exercises

☐ Cough (unsupported)

☐ Supported coughing

☐ Suction

☐ Education

☐ Cycle pedals

☐ Cough assist

Q23. What factors do you take into account when prescribing interventions?

	Never	Rarely	Sometimes	Often	Always
Physician instructions	○	○	○	○	○
O2 requirements	○	○	○	○	○
Rate of Perceived Exertion (RPE) Score	○	○	○	○	○
Discharge status	○	○	○	○	○
Respiratory rate	○	○	○	○	○
SpO2 level	○	○	○	○	○
ABGs	○	○	○	○	○
Gut feel	○	○	○	○	○
Blood pressure	○	○	○	○	○
Heart rate	○	○	○	○	○
Chest x-ray	○	○	○	○	○
Assistance required (mobility)	○	○	○	○	○
Availability of equipment	○	○	○	○	○
Availability of staff	○	○	○	○	○
Patient appearance	○	○	○	○	○
Anxiety level	○	○	○	○	○
BORG score	○	○	○	○	○

Pain (VAS)	○	○	○	○	○
Mobility/functional status	○	○	○	○	○
Other(s) (please specify) ______	○	○	○	○	○

Q24. What components of **breathing exercises** do you incorporate into your post-operative treatment?

	Never	Rarely	Sometimes	Often	Always
Inspiratory hold	○	○	○	○	○
Sustained Maximal Inspiration (SMI)	○	○	○	○	○
Huff +/- cough	○	○	○	○	○
Positive Expiratory Pressure (PEP)	○	○	○	○	○
Incentive Spirometry (IS)	○	○	○	○	○
Thoracic Expansion Exercises (TEE)	○	○	○	○	○
Breathing control	○	○	○	○	○
CPAP (Continuous Positive Airway Pressure)	○	○	○	○	○
IPPB (Intermittent Positive Pressure Breathing)	○	○	○	○	○
Proprioceptive facilitation/input for chest expansion	○	○	○	○	○
Positioning	○	○	○	○	○
Rib springing concept	○	○	○	○	○
Pursed lip breathing	○	○	○	○	○

(PLB)

Other(s) (please specify) ___ ○ ○ ○ ○ ○

Q25. Of the breathing exercise components listed below, rank from 1 to 3 the components you most commonly use on a standard post-op patient.
(Rank where 1 is the most commonly used intervention)

- [ ] Inspiratory hold
- [ ] Sustained Maximal Inspiration (SMI)
- [ ] Huff +/- cough
- [ ] Positive Expiratory Pressure (PEP)
- [ ] Incentive Spirometry (IS)
- [ ] Thoracic Expansion Exercises (TEE)
- [ ] Breathing control
- [ ] CPAP (Continuous Positive Airway Pressure)
- [ ] IPPB (Intermittent Positive Pressure Breathing)
- [ ] Proprioceptive facilitation/input for chest expansion
- [ ] Positioning
- [ ] Rib springing concept
- [ ] Pursed Lip Breathing (PLB)
- [ ] Other (please specify) ___

Q26. Do you prescribe independent breathing exercises for your patients?

Never Rarely Sometimes Often Always

Q27. If yes, what is your prescription of breathing exercises?

Once every waking hour

Every physiotherapy treatment session

As often as patient believes they can manage

Other(s) (please specify)

Q28. What **outcome measures** do you use to monitor the effectiveness of your interventions?

	Never	Rarely	Sometimes	Often	Always
FiO2/O2 requirements	○	○	○	○	○
Respiratory rate	○	○	○	○	○
SpO2	○	○	○	○	○
ABGs	○	○	○	○	○
Progression of assistance required	○	○	○	○	○
Blood pressure	○	○	○	○	○
Heart rate	○	○	○	○	○
Chest x-ray	○	○	○	○	○
Clinical Pulmonary Infection Score (CPIS)	○	○	○	○	○
Sputum clearance	○	○	○	○	○
Distance mobilised	○	○	○	○	○

Readiness for discharge	○	○	○	○	○
Rate of Perceived Exertion (RPE)	○	○	○	○	○
BORG score	○	○	○	○	○
Pain (VAS)	○	○	○	○	○
Anxiety level	○	○	○	○	○
Patient appearance	○	○	○	○	○
Auscultation	○	○	○	○	○
Other(s) (please specify) ______	○	○	○	○	○

Q29. Of the **outcome measures** listed below, rank from 1 to 3 the OCM you most commonly use on a standard post-op patient.
(Rank where 1 is the most commonly used intervention)

☐ FiO2/O2 requirements

☐ Level of mechanical ventilation

☐ VO2 max

☐ Respiratory rate

☐ SpO2

☐ ABGs

☐ Progression of assistance required

☐ Blood pressure

☐ Heart rate

☐ Chest x-ray

☐ Clinical Pulmonary Infection Score (CPIS)

☐ Sputum clearance

☐ Distance mobilised

☐ Readiness for discharge

☐ Rate of Perceived Exertion (RPE)

☐ BORG score

☐ Pain (VAS)

☐ Anxiety level

☐ Patient appearance

☐ Auscultation

☐ Other(s) (please specify) ________

Mobility Prescription Following UAS

Q30. Is it part of your role as a physiotherapist to prescribe **mobility programs** to patients following UAS?

Never	Rarely	Sometimes	Often	Always

Q31. If yes, are you prescribing mobility with the intention of someone else carrying it out?

Never Rarely Sometimes Often Always

Q32. Who carries out the mobility programs that you prescribe? *Tick all that apply.*

Medical staff

Nurse

Physiotherapy assistant

Physiotherapy student

Patient relative/s

Nursing assistant/ patient care assistant

Other(s) (please specify)

Q33. Do you record any of the following patient information in regards to mobilisation? *Tick all that apply*

Day patient first ambulates from the bedside | Length of time physiotherapist spent mobilising the patient | N/A

Q34. Do you believe the above factors listed in the above question (Q32) predict/ dictate patient outcomes?

Yes No

Q35. Why?

Q36. What are the **aims** of mobilisation in patients on each consecutive day following UAS? *Tick one option per day*

	DAY 1

	Never	Rarely	Sometimes	Often	Always	Never	Rar[illegible]
Increased V/Q ratio	○	○	○	○	○	○	○
Improved airway clearance	○	○	○	○	○	○	○
Increased lung volumes	○	○	○	○	○	○	○
Prevention of atelectasis	○	○	○	○	○	○	○
Improved patient mood	○	○	○	○	○	○	○
Improved circulation	○	○	○	○	○	○	○
Fulfill hospital protocols	○	○	○	○	○	○	○
Allow patient to be discharged	○	○	○	○	○	○	○
Improved exercise tolerance	○	○	○	○	○	○	○
Improved bladder and bowel function	○	○	○	○	○	○	○
Normalise blood pressure	○	○	○	○	○	○	○
Prevention of pneumonia	○	○	○	○	○	○	○
Prevention of PPC	○	○	○	○	○	○	○
Other(s) (please specify)	○	○	○	○	○	○	○

Q37. What are the **milestones** you expect patients to achieve on each consecutive day following UAS? *Tick all that apply.*

Sitting in bed, bed exercises: including rolling, bridging, active exercises, cycle ergometry and active assisted exercises.	Day 0	Day 1	Day2	Day3-5
Patient passively moved to chair (no standing) using a hoist, passive lift or slide transfer	Day 0	Day 1	Day2	Day3-5
Sitting over edge of bed (+/- assistance)	Day 0	Day 1	Day2	Day3-5

Transferring bed to chair	Day 0	Day 1	Day2	Day3-5
Marching on the spot (at bedside)	Day 0	Day 1	Day2	Day3-5
Walking away from the bed (5+m) with assistance of 1 person	Day 0	Day 1	Day2	Day3-5
Walking independently with a gait aid away from the bed (5+m)	Day 0	Day 1	Day2	Day3-5
Walking independently without a gait aid away from the bed (5+m)	Day 0	Day 1	Day2	Day3-5
Stairs	Day 0	Day 1	Day2	Day3-5
Other(s) (please specify):	Day 0	Day 1	Day2	Day3-5

Frequency, Intensity and Duration of Mobility Prescription

Q38. How **frequently** would patients undergo **structured mobility** (*mobility prescribed by physiotherapist based on assessment*) on day one (1), day two (2) and day three to five (3-5) post-operatively? *Tick one option per day*.

	Never	Rarely	Sometimes	Often	Always
Day one (1)	○	○	○	○	○
Day two (2)	○	○	○	○	○
Day three to five (3-5)	○	○	○	○	○

Q39. What **determines** the **frequency** of structured patient mobility provided by physiotherapists on each consecutive day following UAS?

	Never	Rarely	Sometimes	Often	Always

Staff availability	○	○	○	○	○
Patient condition	○	○	○	○	○
Pressure to discharge	○	○	○	○	○
Equipment availability	○	○	○	○	○
Physician instruction	○	○	○	○	○
Patient compliance with physiotherapy	○	○	○	○	○
Other(s) (please specify) ____	○	○	○	○	○

Q40. What information do you use to guide your decision making in regards to the **frequency** of your mobility prescription?

	Never	Rarely	Sometimes	Often	Always
Respiratory rate	○	○	○	○	○
Level of ventilation	○	○	○	○	○
Blood pressure	○	○	○	○	○
Readiness for discharge	○	○	○	○	○
SpO2	○	○	○	○	○
Exercise test	○	○	○	○	○
Heart rate	○	○	○	○	○
VO2 max	○	○	○	○	○
BORG	○	○	○	○	○
FiO2	○	○	○	○	○
Arterial Blood Gases	○	○	○	○	○
Evidence/ literature (please specify) ____	○	○	○	○	○

Other(s) (please specify) ☐ ○ ○ ○ ○ ○

Q41. Of the objective measures listed, please **rank** the components you most commonly use to prescribe the **frequency** of your mobility program. (*Rank from 1 to 3, with 1 being the most common.)*

☐ Respiratory rate

☐ Level of ventilation

☐ Blood pressure

☐ Readiness for discharge

☐ SpO2

☐ Exercise test

☐ Heart rate

☐ VO2 max

☐ BORG

☐ FiO2

☐ Arterial Blood Gases

☐ Evidence/ literature (please specify)

☐ Other(s) (please specify)

Q42. What information do you use to guide your decision making in regards to the

intensity of your mobility prescription?

	Never	Rarely	Sometimes	Often	Always
Respiratory rate	○	○	○	○	○
Readiness for discharge	○	○	○	○	○
Level of ventilation	○	○	○	○	○
SpO2	○	○	○	○	○
Exercise test	○	○	○	○	○
Heart rate	○	○	○	○	○
VO2 max	○	○	○	○	○
Blood pressure	○	○	○	○	○
BORG	○	○	○	○	○
FiO2	○	○	○	○	○
Arterial Blood Gases	○	○	○	○	○
Evidence/ literature (please specify) ______	○	○	○	○	○
Other(s) (please specify) ______	○	○	○	○	○

Q43. Of the objective measures listed, please **rank** the components you most commonly use to prescribe the **intensity** of your mobility program. (*Rank from 1 to 3, with 1 being the most common.)*

☐ Respiratory rate

☐ Readiness for discharge

☐ Level of ventilation

- [ ] SpO2
- [ ] Exercise test
- [ ] Heart rate
- [ ] VO2 max
- [ ] Blood pressure
- [ ] BORG
- [ ] FiO2
- [ ] Arterial Blood Gases
- [ ] Evidence/ literature (please specify) ______
- [ ] Other(s) (please specify) ______

Q44. What information do you use to guide your decision making in regards to the **duration** of your mobility prescription?

	Never	Rarely	Sometimes	Often	Always
Respiratory rate	○	○	○	○	○
Readiness for discharge	○	○	○	○	○
Level of ventilation	○	○	○	○	○
SpO2	○	○	○	○	○
Exercise test	○	○	○	○	○
Heart rate	○	○	○	○	○
VO2 max	○	○	○	○	○
Blood pressure	○	○	○	○	○

BORG	○	○	○	○	○
FiO2	○	○	○	○	○
Evidence/ literature (please specify) ☐	○	○	○	○	○
Other(s) (please specify) ☐	○	○	○	○	○

Q45. Of the **objective measures** listed, please **rank** the components you most commonly use to **prescribe** the **duration** of your mobility program. (*Rank from 1 to 3, with 1 being the most common.)*

☐ Respiratory rate

☐ Readiness for discharge

☐ Level of ventilation

☐ SpO2

☐ Exercise test

☐ Heart rate

☐ VO2 max

☐ Blood pressure

☐ FiO2

☐ Arterial blood gases

☐ BORG

☐ Evidence/ literature (please specify) ________

☐ Other(s) (please specify) ________

Q46. What factors do you consider when judging a patient's ability to progress exercise in order to achieve patient milestones?

	Never	Rarely	Sometimes	Often	Always
Physician request	○	○	○	○	○
Gut feeling	○	○	○	○	○
Level of ventilation	○	○	○	○	○
SpO2	○	○	○	○	○
Assistance required	○	○	○	○	○
Heart rate	○	○	○	○	○
VO2 max	○	○	○	○	○
Blood pressure	○	○	○	○	○
BORG	○	○	○	○	○
FiO2	○	○	○	○	○
Arterial Blood Gases	○	○	○	○	○
Pain (VAS)	○	○	○	○	○
Respiratory rate	○	○	○	○	○
Availability of staff	○	○	○	○	○
Evidence/ literature (please specify) ________	○	○	○	○	○
Patient able to ambulate length of corridor	○	○	○	○	○
Other(s) (please specify)					

Q47. What, if anything, do you monitor during mobilisation of UAS patients? *Tick all that apply*

Respiratory rate

Gut feeling

Readiness for discharge

Level of ventilation

SpO2

Exercise test

Assistance required

Pain (VAS)

Availability of equipment

Patient appearance

Other(s) (please specify)

Q48. What **outcome measures** do you most commonly use to determine the effectiveness of mobility prescription in UAS patients? (*Rank from 1 to 3, with 1 being the most commonly used.)*

Readiness for discharge

Level of ventilation

Blood pressure

Rate of perceived exertion

FiO2

Heart rate

BORG

VO2 max

Chest x-ray

Pain (VAS)

Respiratory rate

Clinical Pulmonary infection Score

Anxiety level

SpO2

Sputum clearance

Patient appearance

ABGs

Distance mobilised

O2 requirements

☐ Progression of assistance required

☐ Walk test (2 minute, 6 minute, 10m self paced walk test, 40m self paced walk test, 20m self paced walk test)

☐ Functional mobility index ☐ De Morton Mobility Index (DEMMI)

☐ Timed Up and Go (TUG)

☐ Other(s) (please specify) __________

Discharge Planning

Q49. What are your discharge criteria for UAS patients from physiotherapy services? *Tick all that apply*

Functional independence

Home exercise program

No longer physiologically deranged

Adequate cough

Able to climb stairs

Distance mobilised (please specify) __________

Physician instruction (please specify) __________

Other (please specify) __________

Q50. On average, what day post-operatively do you usually stop seeing your patients from a physiotherapy perspective?

Day 1

Day 2

Day 3

Day 4

Day 5

Other (please specify)

Q51. Do you ever refer your patients for follow up physiotherapy management following discharge?

Never   Rarely   Sometimes   Often   Always

Q52. If yes, where do you refer your patients? *Tick all that apply*

General Practitioner

Physiotherapy private practitioner

Hospital outpatient department

Community physiotherapy services

Falls management clinics

Other (please specify)

Q53. Do you supply patients with a mobility program to follow upon discharge?

Never   Rarely   Sometimes   Often   Always

Q54. What do you typically include in your mobility program?

Q55. If you wish to make any additional comments, please write them below.
